# Affective trajectories: Are hens influenced by positive and negative changes in their living conditions?

**DOI:** 10.1016/j.applanim.2023.105883

**Published:** 2023-03-10

**Authors:** Elizabeth S. Paul, William Browne, Michael T. Mendl, Gina Caplen, Suzanne Held, Anna Trevarthen, Christine J. Nicol

**Affiliations:** a Bristol Veterinary School, University of Bristol, Langford House, Langford BS40 5DU, UK; b School of Education, University of Bristol, 35 Berkeley Square, Bristol BS8 1JA, UK; c Royal Veterinary School, Hawkshead Lane, Brookmans Park, Hatfield AL9 7TA, UK

**Keywords:** Chicken, Affect, Trajectory, Judgement bias, Cognitive bias, Welfare

## Abstract

Most studies of the effects of housing and husbandry on animals’ affective states and welfare investigate the impact of *stable* living conditions, comparing for example, animals living in enriched environments with those living in non-enriched ones. *Changes* in living conditions, including from more to less enriched environments, have also been found to have effects on measures of affective state and welfare in some species. But these studies have not investigated whether it is the *trajectory of change* that has affected the animals (e.g., worsening conditions), or simply the nature of their *final environment* (e.g., non-enriched). Here, we hypothesised that laying hens living in worsening conditions across a six-week period (gradually moving from preferred to non-preferred living conditions; “Trajectory to Non-Preferred”, TNP, n = 30), would show evidence of more negative affective states and poorer welfare than those living continuously in non-preferred conditions for the same duration (“Stable Non-Preferred”, SNP, n = 30). We also hypothesised that hens living in improving conditions (gradually moving from non-preferred to preferred living conditions; “Trajectory to Preferred”, TP, n = 30), would show evidence of more positive affective states and better welfare than those living continuously in preferred conditions (“Stable Preferred”, SP, n = 30). The preferred living condition provided extensive resources and intermittent rewarding events (such as the delivery of food treats) known to be valued and preferred by most hens, while the non-preferred living condition provided just basic resources and intermittent aversive events (e.g., loud noises). The hens’ affective states and welfare were measured using home-pen behavioural observations, body condition assessments, physiological stress measures (e.g., blood corticosterone, glucose, etc.), physical challenge tests, and judgement bias tests. A number of differences between hens in the trajectory and stable living conditions were found: TP hens were lighter, showed more foraging behaviour and less standing alert and head-shaking than SP hens, while TNP hens showed more head-shaking, mild feather pecking and aggressive attacking of pen mates than SNP hens. However, some of these differences failed to reach significance following Benjamini adjustments for multiple testing. The groups also did not differ in their judgement biases (measured in a sub-sample of 12 hens per experimental group), response to physical challenges, or measures of physiological stress. We conclude that the hens in the present study showed some evidence of responsiveness to ‘affective trajectories’ in their living conditions, but no definitive effects on their affective states and welfare.

## 1 Introduction

In contemporary animal welfare research, whether living conditions are regarded as good, adequate, or poor, depends in large part on evidence regarding the preferences that animals have demonstrated experimentally (e.g., Dawkins, 1990; Dawkins, 2015; Mason et al., 2001; Mendl et al., 2017). Domestic chickens generally express preferences for, and willingness to expend energetic resources to obtain: More space (Lindberg and Nicol, 1996), enclosed nest boxes (Appleby and McRae, 1986; Cooper and Appleby, 1996; Cooper and Appleby, 2003; Krusch-witz et al., 2008; Struelens et al., 2008a), high perches (Olsson and Keeling, 2002; Schrader and Muller, 2009; Struelens et al., 2008a), deep litter floor substrates (Dawkins and Beardsley, 1986), and fine-grained dustbathing substrates (Petherick and Duncan, 1989; Wichman and Keeling, 2009; Widowski and Duncan, 2000). Chickens have also been found to prefer environments in which background noise levels are low (Jones, 1986; Jones et al., 2012; Mackenzie et al., 1993; McAdie et al., 1993), and where they have access to high and low light intensity areas (e.g., a canopy; Bright et al., 2011; Davis et al., 1999). According to this preference-based approach, generally-preferred, captive living environments (in which all of an animal’s needs and desires are catered for) might reasonably be expected to maximise positive affect and welfare. However, while this may be the case regarding much of an animal’s physical condition and general health status, views of welfare that take account of the “subjective” states of animals (Browning, 2020; Dawkins, 2015; Duncan, 1996; Mason and Mendl, 1993; Mendl et al., 2017; Paul et al., 2020), must consider an additional possibility: that the experience of environmental *change*, from less to more preferred, or from more to less preferred, might also have an important impact on affective states and thereby, on welfare.

The hypothesis that improving and worsening living conditions might have stronger influences on animals’ affective states than stable good (preferred) and bad (non-preferred) ones alone, has origins in both the theoretical biology of animal affect, and empirical studies of humans’ self-reported affect. From a biological and adaptive point of view, it is argued that animals’ affective states can be used to represent information about the world they live in, functioning as aids to decision-making (Eldar et al., 2021; Gygax, 2017; Mendl et al., 2010; Mendl and Paul, 2020; Nettle and Bateson, 2012; Trimmer et al., 2013). This can be seen as a kind of meta-learning: one’s past experience of a wide range of rewarding and punishing stimuli feed down into a single set of affectively-based predictions - the likelihood of positive (rewarding) events occurring and the likelihood of negative (threatening, punishing) events occurring in the present moment. From this base, it is only a small step to hypothesise an additional layer of meta-learning: that one is living in *improving* or *worsening* conditions (Eldar et al., 2016, 2021). Such improving and worsening environments are likely to be common in nature. For example, the approach of summer heralds increasingly available rewards (e.g., food, mates), as well as improvements in other preferred conditions (e.g., warmer temperatures). A predator boom, on the other hand, might see threat to prey species increase exponentially, ramping up the probability of harm and death. And similarly, a drought might gradually reduce vegetation cover, increasing the visibility and accessibility of prey species to predators. If meta-learning about the overall levels of reward and punishment in an environment can occur, then it may well be adaptive for learning about *trajectories* of reward and punishment to also occur.

Evidence from psychological studies show that humans’ affective states are particularly strongly associated with *changes* in rewarding and punishing experiences. Prominent among these are findings regarding people’s responses to unexpected gains and losses (Rutledge et al., 2014, 2015), and to life events that shift people’s physical and/or economic circumstances considerably (e.g. Brickman and Campbell, 1971; Brickman et al., 1978; Gilbert, 2009). While there is some complexity to these results (see Kettlewell et al., 2020), the broad findings are clear: rewarding and punishing changes in people’s lives can strongly influence affective states and well-being, especially over short-to-medium time-periods. However, in the longer-term, such shifts into more or less preferred circumstances have surprisingly small effects on self-reported ratings of happiness. In sum, it seems that people’s affective systems tend to adapt (e.g., to being a millionaire or a paraplegic; for reviews see (Diener et al., 2006; Frederick and Loewenstein, 1999; Luhmann et al., 2012). At the societal level, changes in national economic circumstances appear to have similar effects: across periods of one or two years, recessions and economic growth influence the mean happiness of populations, but over longer periods, the effects of countries’ ongoing economic circumstances are small (Easterlin, 2015). In sum, people’s affective systems, and their self-reported happiness levels, are particularly strongly affected by changes in circumstance, especially if these changes are sudden, or occur over relatively short periods of time.

To date, the question of whether non-human animals are influenced by improving or worsening living conditions has not been investigated, even though it is well known that some species are affectively responsive to abrupt shifts in the value of individual rewards (e.g., (Papini and Dudley, 1997). In animal welfare research, a number of studies have made use of environmental change (e.g. from enriched to non-enriched living conditions) as an affect manipulation (e.g., Barker et al., 2017; Bateson and Matheson, 2007; Brydges et al., 2011; Burman et al., 2008a; Burman et al., 2009; Douglas et al., 2012), perhaps with the presumption that this change will have a greater impact than stable (e.g. non-enriched) conditions alone. But this presumption has not, to our knowledge, been tested experimentally.

**Fig. 1:**
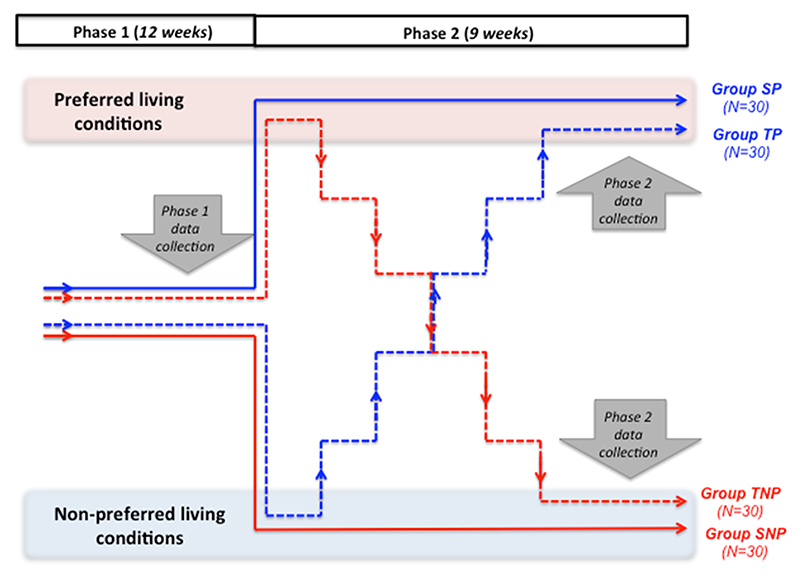
Graphical illustration of study design (not to scale). Note that during the two data collection periods, both groups SP and TP are in the same living conditions (intermediate and preferred, respectively), as are groups SNP and TNP (Intermediate and Non-Preferred, respectively).

Here, we investigated the effects of environmental changes on the affective state and welfare of an important and highly populous domestic species, the chicken (*Gallus gallus domesticus*). Our aim was to discover whether birds experiencing improving (i.e. increasingly preferred, “positive trajectory”) living conditions would show signs of more positive affect and better welfare than those experiencing stable, preferred conditions, and whether worsening (i.e., increasingly less-preferred, “negative trajectory”) living conditions would give rise to poorer affect and welfare than stable, non-preferred ones (Fig. 1).

## 2 Materials and methods

### 2.1 Ethical note

All work was approved by the University of Bristol Animal Welfare and Ethical Review Body and conducted under U.K. Home Office Licences (PPL: 30/2779 and 30/3392). Animal use and care was in accordance with the Animals (Scientific Procedures) Act 1986, EU directive 2010/63/EU and UK Home Office code of practice for the housing and care of animals bred, supplied or used for scientific purposes. All husbandry and experimental procedures were designed to avoid and minimise distress, with procedures in place to terminate any task or measurement in the event of a hen becoming agitated or fearful. Animals were regularly weighed and the birds’ general health and wellbeing were monitored daily by professional animal caretakers.

### 2.2 Subject birds

Subject birds were 120 medium-brown commercial laying hens (British blacktail hybrids), sourced from a local commercial breeder in two separate batches (60 birds per batch, approximately 1 year apart), at 18 weeks of age. They were all raised in standard rearing conditions (although unfortunately, full details of these are not available) and all had been beak-trimmed. On arrival at the laboratory all birds were wormed, treated prophylactically for mites, and individually marked with coloured leg bands and a patch of coloured stock-marker spray paint. They were immediately randomly allocated to pens of five birds per pen and remained in these social groups for the duration of the study. Throughout the study, all birds received the same ad libitum feeding regime (Farmgate Layers’ Mash, BOCM Pauls, Ipswich, Suffolk, U.K.). They were kept on a 12-hour light-dark cycle (light period 07:00–19:00 h) with an ambient temperature of 19–21 °C. Eggs were collected daily and pens were cleaned weekly.

Each pen of birds was allocated to one of four experimental groups: SP (Stable Preferred), TP (Trajectory to Preferred), SNP (Stable Non-Preferred) and TNP (Trajectory to Preferred). These four groups (30 birds and 6 pens per group) were evenly balanced across the two years’ batches of hens. At the end of the study, all 60 TP and TNP birds were rehomed as pets or as hobbyist small-holder stock. The 60 hens belonging to Groups SP and SNP were kept in the laboratory to complete a sister study – a continuing experiment comparing their long-term responses to (stable) preferred and non-preferred living conditions across 24 weeks of exposure (Paul et al., 2022). The results of that study confirmed that the P condition was preferred, and the NP condition not preferred, by the majority of those birds. Note that some of the data presented here for Groups SP and SNP were also used in this longer-term study (see (Paul et al., 2022).

### 2.3 Experimental design

The study took place across two phases (see Fig. 1). Phase 1 was an initial 12-week period during which all birds were kept in (stable) “Intermediate” living conditions (see details of this and other living conditions below). These living conditions were called Intermediate because they represented an approximate midpoint between the two experimental living conditions, P (Preferred) and NP (Non-Preferred) (although note that this did not necessarily represent a ‘midpoint’ for the hens). Because Phase 1 took place within these Intermediate living conditions, all birds in the study experienced the same environments during initial training and testing, making their data from this phase fully comparable. An alternative design would have been to keep both SNP and TNP birds in Non-Preferred conditions for the whole of Phase 1, and both SP and TP birds in Preferred conditions for the whole of Phase 1, but this was not possible for a range of practical reasons (see also Paul et al., 2022).

A subset of the birds in each pen (40 %; 2 birds per pen) were trained to complete a Go/No-go screen-peck judgement bias task during the Phase 1 period. These birds were selected randomly from each pen, following initial screening to exclude any birds that were unable or unwilling to eat mealworms or that did not respond to initial clicker training. In the final two weeks of Phase 1, these birds undertook judgement bias testing, and a comprehensive range of baseline data were collected from these and all other birds in the study (behaviour in home pen; behavioural tests; physiological measures – see Section 2.4. below). Evidence for designing the P and NP living conditions came from published studies that found that chickens had preferences for particular environments or resources over others, and/or that demonstrated that most chickens would work to obtain access to them. Non-Preferred: Background noise and loud sounds (Jones et al., 2012; Mackenzie et al., 1993; McAdie et al., 1993); Discrete punishers (Jones, 1986). Preferred: Dustbath and dustbathing substrates (Petherick and Duncan, 1989; Vanliere, Kooijman, & Wiepkema, 1990; Wichman and Keeling, 2009; Widowski and Duncan, 2000; Discrete rewards (Bouvarel et al., 2009; Bruce et al., 2003; Moe et al., 2013); Floor substrates (Dawkins and Beardsley, 1986); Shade canopy & light intensity variation (Bright et al., 2011; Davis et al., 1999); Nestbox design (Appleby and McRae, 1986; Cooper and Appleby, 1996; Cooper and Appleby, 2003; Freire et al., 1997; Hughes, 1993; Kruschwitz et al., 2008; Reed and Nicol, 1992; Struelens et al., 2008b); Perch design (Appleby and Hughes, 1995; Chen et al., 2014; Olsson and Keeling, 2002; Pickel et al., 2010; Reed and Nicol, 1992; Struelens et al., 2008b); Space allocation and stocking density (Reed and Nicol, 1992).

When Phase 1 data collection was complete, the hens were moved to their new experimental living conditions for Phase 2, while remaining with the same group of 5 birds they had lived with in Phase 1. Full details of the living conditions of birds in each group are given in Table 1. The P living condition comprised many preferred resources and rewards, based on previous published studies of hens’ expressed preferences. (see (Paul et al., 2022), p175, for full details). Similarly, the NP living condition comprised many non-preferred resources and punishers.

Experimental groups SP and SNP were kept in these conditions, respectively, for the whole of Phase 2. The trajectory group TP had living conditions altered gradually across Phase 2, on a weekly basis (with successive stages marked as NP, NP1, NP2, P2, P1, P; while group TNP experienced equivalent alterations in the opposite direction P, P1, P2, NP2, NP1, NP in Table 1). The conditions of the TP hens were designed to improve, while those of the TNP birds were designed to worsen. Housing design and furnishings, light/shade, ambient noise and daily, quantified exposure to positive and negative events were all manipulated across the six weeks of Phase 2 for these birds.

To minimise disruption to the birds, weekly shifts in living conditions for groups TP and TNP coincided with weekly cleaning sessions; birds were removed from their pens and placed in crates while cleaning and changes took place (approx. 30 min). Non-trajectory birds were crated and cleaned in parallel with these. After the 6-week trajectory period, TP hens had completed their shift from Non-Preferred (NP) to Preferred (P) living conditions, and TNP hens had completed their shift from Preferred (P) to Non-Preferred (NP) living conditions. There then followed a second period of data collection (one week of judgement bias refresher-training and then two further weeks of data collection as in Phase 1), during which time they remained in these same, final living conditions. Birds that had been in the stable groups SP and SNP remained in these conditions and underwent the same three weeks of Phase 2 data collection.

### 2.4 Data collection

Data collection took place in two, two-week phases, with an additional week for judgement bias re-training at the start of the Phase 2 data collection period. Test data were collected in two separate ‘clinics’ – Clinic A (blood/faecal testing and physical examination) was conducted in the mornings of the data collection period and Clinic B in the afternoons (physical challenge tests: High Perch test and Water Box test). Half of the birds underwent Clinic A first, and half underwent Clinic B first (balanced for experimental group). Behavioural observations, judgement bias testing and the Mealworm test (an adjunct to judgement bias testing) were conducted on alternating, non-clinic days.

A large number of measures (physical, physiological, behavioural, behavioural tests) were taken for the purposes of gauging the affective states and welfare of subject hens. The selection of these ‘welfare indicators’ was based on a number of pragmatic and theoretical reasons: successful use in prior studies, previous associations with measured preference, quick and efficient to obtain from large numbers of birds (Nicol et al., 2011; Nicol et al., 2009; Paul et al., 2022; Welfare Quality, 2019). Some of the measures used are commonly employed in studies of welfare (e.g. body condition score, body mass, comb and foot lesions), while others are more novel. For full details of the physical, physiological and behaviour-test data collected, and information regarding their expected relationships to affective state and welfare, please see Appendix A (Supporting Information).

#### 2.4.1 Weighing and physical examination

Hens were taken individually to an experimental room adjacent to their pens for weighing and physical examination. Body condition was scored as a binary variable according to whether it was considered (1) “good” - the breast was plump (no keel bone detectable) or (0) “poor” - the breast was thin (keel bone detectable) under gentle palpation (Gregory and Robins, 1998). The presence/absence of comb and foot lesions was recorded, and claw length was measured (middle claw, left foot; mm). Binary classifications were also used to record comb elevation (0: upright; 1: floppy), comb colour (0: bright; 1: pale), and comb size (0: small, <3.5 cm^2^; 1 = large, >3.5 cm^2^).

#### 2.4.2 Blood and faecal testing

Blood sampling was conducted in an experimental room adjacent to the hens’ home pens (sound-isolated by two doors). Birds were removed individually to minimise disruption and stress; it was our aim to minimise the time taken between catching and blood sampling. We achieved this by using separate staff members to catch birds and to take the blood sample, and by placing birds in crates with their pen mates following sampling to recover prior to returning to the home pen. A total of 4 ml of blood was collected from each bird’s wing vein using a 23-gauge 16 mm needle. It was then split between a Serum Collection Vacutainer tube and an EDTA Blood Collection Vacutainer tube, and put into ice storage for subsequent analyses. For approximately two hours following sampling, tested birds were visually assessed to ensure that there were no signs of distress. Samples of whole blood were analysed for glucose and triglyceride levels (Langford Diagnostic Laboratories). Two blood smears were made on glass slides and counts of heterophils (H) and lymphocytes (L) taken, to calculate the H:L ratio (Gross and Siegel, 1983). Blood samples collected for serum extraction were kept at 0 °C overnight and then centrifuged at 1200 rpm for 15 min and the serum stored at – 18°C until analysis for corticosterone (Cambridge Specialist Laboratories).

Faecal samples were collected on the same days as blood sampling. To obtain a faecal sample, each hen was placed in an individual wire cage with a plastic sheet floor, with at least one other hen visible in an adjacent cage. Hens were left quietly until they had produced a faecal deposit (max. 60 mins). Faecal samples were frozen in 50 ml plastic tubes at – 20 °C. When all samples had been collected, they were desiccated using a freeze drier (over three days) to obtain a measure of bulk water content (Puvadolpirod and Thaxton, 2000; Nicol et al., 2009; Nicol et al., 2011).

#### 2.4.3 Behavioural observations in home pen

Videos were taken of all pens for 1 h at two time points (starting at 0700 and 1500 h), across 3 days during each phase’s data collection period (i.e. 6 h of video per phase). It was retrospectively coded by three observers (assigned 1 day each per bird, to avoid systematic observer bias) using a detailed ethogram derived from Appendix 3, p176(Nicol et al., 2009; Paul et al., 2022), and specialist software (Observer XT 10, Noldus, Wageningen, Netherlands). Because the P and NP pens were visibly different, observers could not be blind to the living condition type, although they were blind to the presence or absence of a trajectory manipulation.

Two types of behavioural observations were conducted: Scan observations and Focal observations. Scan observations were used to provide an overview of the birds’ time budgets for all common behaviours (1 % or more of all hens’ time observed: Feed from Hopper, Ground Forage (a compound of Ground Peck and Ground Scratch), Stand Alert, Preen, Walk, Nest, Drink, Sit. The behaviour patterns of each bird were recorded instantaneously at 10 min intervals (7 scans/h) for all 6 h of video recordings per phase. Because the initial scan of each recorded day was at the moment that lights came on (0700 h), it was deleted from the analysis, leaving a total of 13 scan points per day (39 per bird per phase). Scan data reported here are expressed as a percentage of all scans (per bird per phase) in which a behaviour pattern was observed.

Continuous focal observations of each bird made use of the middle 30 min of the same set of videos (0715–0745 h and 1515–1545 h). Behaviours were recorded as average frequencies across all 30 min observation periods per day. These continuously recorded observations were used to analyze the frequencies of social, emotional and aggressive behaviours not commonly observed within the scan sampling regime (Aggressive attack given, Aggressive attack received, Severe feather peck given, Severe feather peck received, Mild feather peck given, Mild feather peck received, Beak peck given, Beak peck received, Head-shake, Dustbathe/Sham Dustbathe).

#### 2.4.4 Judgement bias test

Two hens per pen (12 per experimental group; total n = 48) were judgement bias-trained using the apparatus and methods described by Deakin et al. (2016) (see also Paul et al., 2022). This was a Go/No-go screen peck task in which the hens were trained to peck a positive predictor cue (S+) displayed upon a computer screen to obtain a mealworm reward, and to avoid pecking a negative predictor cue (S-) to avoid receiving an air puff. The cues presented on the screen were 3.5 cm diameter orange coloured circles of high or low saturation (High: Hue=19, Saturation=250, Lightness=19; Low: Hue=19, Saturation=50, Lightness=19); whether the S+ or S- was high saturation was balanced across experimental groups. Three ambiguous probes cues were used: near S-; Middle; near S+ (these had values of Saturation=100, 150, 200, with Hue and Lightness remaining constant at 19). Four test sessions (one per day) were performed at the end of each of the two experimental phases; each test session comprised 40 stimuli presentations including 17x S+ cues, 17x S- cues and 6x ambiguous cues (2 of each type). The order in which the ambiguous probe cues were presented was the same for all birds but differed across the test sessions so that all birds saw four different sequences.

The main summary variables derived from these judgement bias tests were “Proportion of ambiguous probes pecked” and “Mean latencies to peck ambiguous probes”. These are generally regarded as measures of an animal’s propensity for optimistic-like or pessimistic-like responses to ambiguity (i.e., their expected probability of reward or punishment, and/or their willingness to risk reward loss or punishment). As a way of checking to make sure that the tests were not simply measuring the birds’ valuation of the mealworm food reward, an additional test of total mealworm consumption in a 1-minute period was also performed. For this Mealworm test, each bird was placed on the floor of the test room and given a ceramic bowl containing 100 g of live mealworms. The mass remaining after 1 min was used to calculate the mass of mealworms consumed per minute. Two Mealworm tests were conducted on sequential days, giving a measure of mean mass consumed per test. An additional score of mass consumed as proportion of the hen’s body weight was also calculated.

#### 2.4.5 Physical challenge tests

Two tests were used as measures of hens’ responses to physical challenges; in essence, these were decision-tasks that required the hens to make stay/go choices based on assessments of their own physical capacity. We assessed physical challenge in two contexts, the first positive (food reward), and the second negative (water punisher).

The High Perch test considered hens’ willingness to jump down to the floor, from a 1 m high perch, for a six-mealworm reward. Mean latencies to jump across three successive tests were calculated (maximum duration of test, 180 s). It has previously been found that the ability of hens to jump for food is compromised if they are in poor physical condition or are in pain (Nasr et al., 2012, 2015). Full details of the High Perch test are given in (Paul et al., 2022).

The Water Box test assessed the latency of hens to jump out of a 6 cm deep plastic box containing shallow, tepid, water (1 cm), across three successive tests (maximum duration of test: 180 s). The median latency to exit the water box across all hens in Phase 1 testing, was 10 s. This was used to generate a categorical measure of willingness to jump (<= 10 s; >10 s). It has previously been found that birds in poor physical condition (e.g., broilers suffering from lameness) are slower to avoid or escape contact with water (Weeks et al., 2002). Although we are not aware of this test previously being used with laying hens, its association with poor physical condition and pain point to its likely value in welfare assessment of all chickens (e.g., see (Casey-Trott and Widowski, 2016). Full details of the Water Box test are given in (Paul et al., 2022).

### 2.5 Data analysis

Following preliminary analyses of the judgement bias data to confirm that the task had been learned correctly, two main sets of analyses were conducted: Comparison of Group P and Group TP birds (stable preferred and trajectory-to-preferred living conditions) and comparison of Group SNP and Group TNP birds (Stable Non-Preferred and Trajectory-to-Non-Preferred living conditions). We hypothesised that experiencing trajectories of living conditions, comprising increasingly preferred or non-preferred components, would influence hens’ affective and welfare indicators in ways that differed from those seen in hens in identical but stable environments. Multi-level models, with hens nested in pens, were used. To control for existing individual variation, data taken at the end of Phase 2 were adjusted for the measures taken at the end of Phase 1 (i.e. baseline).

For continuous welfare indicator variables (including the Judgement Bias test data – Proportion of probes pecked) that could be considered normally distributed, models of the following form were used:

$$WI_{ij}=\alpha +\beta {Group}_{ij}+\gamma {WIbase}_{ij}+u_{j}+e_{ij}$$

Here, *WI*_*ij*_ is the value of the welfare indicator for hen *i* in pen *j* at the later time point (Phase 2), *Group*_*ij*_ indicates the experimental group for hen *i* in pen *j*, while *WIbase*_*ij*_ is the value of the welfare indicator for hen *i* in pen *j* measured in Phase 1 (baseline). The intercept is given by *α*, while *γ* provides an estimate of the strength of relationship between the two time points. The model has random effects, *u*_*j*_ and *e*_*ij*_ for pen and hen respectively, that are assumed to be normally distributed and capture variations due to individual pens and hens. The term of interest was *β*, which captures the difference between each pair of experimental groups, having controlled for Phase 1 (baseline) measures and any clustering due to pens.

For binary welfare indicator variables, equivalent multilevel logistic regression models were fitted:

$$WI_{ij}\sim Bernouilli\left(\pi_{ij}\right),logit\left(\pi_{ij}\right)=\alpha +\beta {Group}_{ij}+\gamma {WIbase}_{ij}+u_{j}$$

Here again, our interest lay in *β*, which captures the differences between each pair of experimental groups, having controlled for Phase 1 (baseline) measures and clustering due to pens.

The number of statistical tests conducted for each of our two main group comparisons (SP vs TP and SNP vs TNP) was large (n = 39). In order to reduce the possibility of Type 1 errors, therefore, we conducted Benjamini false discovery rate (FDR) calculations (Benjamini and Hochberg, 1995; Benjamini and Liu, 1999), generating conservative thresholds for significance, using FDRs of 0.2 and 0.05 (i.e., expectation of false positive results of, at most, 20 % and 5 %, respectively). In the results section below, we present our results both with and without these Benjamini adjustments.

## 3 Results

Of the 120 hens included in the experiment (Group SP (N = 30); Group TP (N = 30); Group SNP (N = 30); Group TNP (N = 30)), one failed to complete the study. This was a judgement-bias trained hen from Group TP. At the end of Phase 1 data collection, the hen started to isolate herself in the pen as a result of aggression from pen mates. She was inspected by a veterinarian who determined that she was otherwise physically fit and well; a decision was made to remove her from the study and she was rehomed successfully. None of her data are included in the present results. Another hen, from Group TNP (not a judgement bias trained bird), was discovered to have a congenital defect, which left her without fully-formed wings. She was otherwise healthy, however, and took part in the whole experiment, apart from the High perch test and the blood tests.

### 3.1 Judgement bias test face validity

For the judgement bias task, 47 hens (Group SP, n = 12; Group SNP, n = 12; Group TP, n = 11; Group TNP, n = 12) were successfully trained and completed the Go/No-go screen peck task for Phase 1 and Phase 2 data collection. Preliminary analyses of all of these birds’ performances on test sessions at the end of the Phase 1 demonstrated that the hens had successfully learned to discriminate the S+ and S- cues; paired t-tests showed that they pecked the coloured disc that predicted reward significantly more frequently than the disc that predicted punishment (Mean percentage of S+ cues pecked = 99.06 %; Mean percentage of S-cues pecked = 1.31 %; t_(46)_ = −225.583, p < 0.001). These birds also showed the expected generalisation across the three intermediate probe cues (Near S+, Middle, Near S-), with the Middle probe being pecked significantly more often than the S- (t_(46)_ = 12.386, p < 0.001) and less often than the S+ (t_(46)_ = −8.381, p < 0.001).

Initially, two summary variables, “Proportion of ambiguous probes pecked” during test sessions (out of a total of 24 probes presented; 8 Near S+, 8 Middle; 8 Near S-), and “Mean latency to peck ambiguous probes” were calculated for all birds. Across the entire sample of judgement bias-trained hens (n = 47), these two variables were strongly inversely correlated (Phase 1, Pearson’s r = −0.987, p < 0.001; Phase 2, Pearson’s r = −0.982, p < 0.001); for the purposes of the presentation of results, therefore, only the first of these have been presented in the results section here.

#### 3.1.1 Colour saturation of S+ and S- cues in judgement bias test

It has previously been noted that judgement bias tasks using the method employed here have been subject to a significant effect of the colour saturation of cues used as the S+ and S- anchors (Paul et al., 2022). Analyses indicated that this was also the case here: Across all 47 birds, 23 of whom had been trained to associate highly saturated coloured discs (orange disc image on computer screen) with the opportunity for reward (mealworm), and 24 of whom had been trained to associate highly saturated coloured discs with the threat of punishment (air puff), there was a significant effect of this colour saturation on both latency to peck all ambiguous probes and the proportion of ambiguous probes pecked (independent sample t-tests: t_(45)_ = 5.089, p < 0.001; t_(45)_ = −5.058, p < 0.001 respectively). The probable explanation for this effect is a perceptual asymmetry of colour saturation, with the “middle” probe being perceived as more similar to the S+ when the S+ is of high a saturation colour, and more similar to the S- when the S- is high saturation (Paul et al., 2022). Because the colour saturation of the S+ and S- was equally distributed across the experimental groups in this study (in a counter-balanced design), however, no confounds arising from this were anticipated.

#### 3.1.2 Judgement bias scores and motivation to feed

Checks were made to establish whether hens’ responses in the Judgement Bias test could be interpreted simply as a reflection of motivation to feed. Correlational analyses were conducted between the main summary variable, Proportion of ambiguous probe cues pecked (Phase 1), and measures potentially associated with motivation to feed: Body mass and meal-worm consumption tests. These did not show any evidence of an association between feeding motivation and judgement bias responses (Body mass r = 0.039, *n.s*.; Mealworm test r = −0.002, *n.s*.; Mealworm test controlling for body mass r = −0.004, *n.s*.).

### 3.2 Comparisons of SP and TP hens

#### 3.2.1 Judgement bias scores (SP and TP hens)

The SP and TP birds did not differ significantly from one another in the Judgement Bias test in the total proportion of ambiguous probe cues they pecked at Phase 2. Further analyses, considering the proportion pecked of each cue separately (i.e. S+, Near S+, Middle, Near S-, S-) also revealed no significant differences between the groups.

#### 3.2.2 Physical measures (SP and TP hens)

Group TP hens were found to be significantly lighter (lower in body mass) than SP hens at the Phase 2 data collection point (i.e. after controlling for body mass in Phase 1: β = 44.03 (SE 17.990), p = 0.017; see Table 2 below). This difference did not remain significant following Benjamini adjustments for multiple comparisons, however (FDR 0.2, and 0.05). The groups did not differ in any of the other physical measures taken (body condition score; foot lesions and cleanliness; claw length; comb elevation, colour and size).

#### 3.2.3 Blood and faecal measures (SP and TP hens)

The values for each group’s blood physiology measures are summarised in Table 2. No significant differences between the experimental groups were found. There were also no differences in faecal bulk water content.

#### 3.2.4 Behavioural measures (SP and TP hens)

**Fig. 2:**
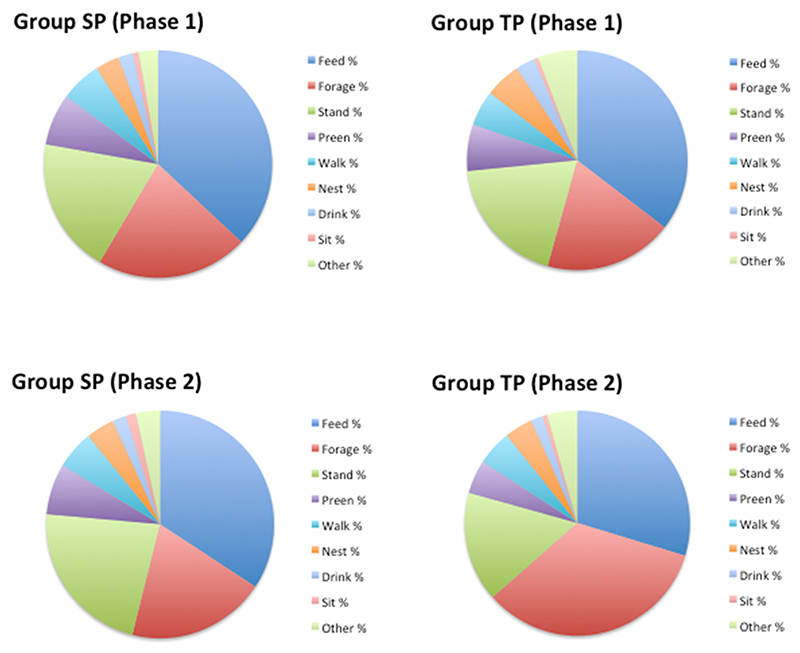
Pie charts illustrating the behavioural time-budgets observed in SP and TP birds, during scan sample observations in Phases 1 and 2.

Behavioural time-budgets of SP and TP birds, obtained from scan observations, are shown in Fig. 2 as mean percentages of all scans made. Feeding (at the feeder), Foraging from the floor of the pen (ground scratching and ground pecking combined), and Standing Alert were the most common behaviours shown by all hens, together contributing to approximately three-quarters of all scans during the daylight hours when observations took place (a.m. and p.m.). Following this came Preening and Walking, while Nesting, Drinking (at the drinker) and Sitting were relatively infrequently observed.

Analyses of these scan observation data revealed a number of significant differences between the groups: by the Phase 2 data collection point, TP birds showed more Foraging behaviour (Ground Peck and Ground Scratch) than SP birds (i.e. a greater increase; β = 15.958 (SE3.515), p < 0.001), less Standing Alert behaviour (β = −6.604 (SE2.250), p = 0.005), and less Preening (β = –2.393 (SE1.016), p = 0.019) when compared with SP birds. These differences remained following Benjamini adjustments using an FDR of 0.2, but only Foraging differed significantly between the groups when an FDR of 0.05 was employed.

Analyses of the frequencies of rarer (predominantly social/affective) behaviours observed during focal observations revealed differences between SP and TP birds in Head-shaking behaviour: 60 % of Group SP and 31 % of Group TP birds were observed Head-shaking once or more across all three days’ 30 min of focal observations (β = 1.202 (SE 0.548), p = 0.028). However, this difference was no longer significant following Benjamini adjustments (FDR 0.2 and 0.05). No group differences were found in Aggressive attacks, Feather pecking, Beak pecking or Dustbathing.

#### 3.2.5 Physical challenge test measures (SP and TP hens)

Group SP and TP birds did not differ in their responses to the High Perch Test or the Water Box Test.

### 3.3 Comparisons of SNP and TNP hens

#### 3.3.1 Judgement bias scores (SNP and TNP hens)

Analyses of hens’ performances in the Judgement bias test were based on the total proportion of ambiguous probe cues pecked during test sessions; no significant differences were found between the groups in this measure at the Phase 2 data collection period. Additional comparisons of the hens’ responses to all cues separately (S+, Near S+, Middle, Near S-, S-) also revealed no significant differences between the groups.

#### 3.3.2 Physical measures (SNP and TNP hens)

No significant weight (body mass) differences between SNP and TNP birds were found at the Phase 2 data collection period (i.e. after controlling for body mass in Phase 1). The claws of SNP birds were significantly longer than those of TNP birds at the Phase 2 data collection point (β = 1.942 (SE 0.280), p < 0.001), and this difference remained significant following Benjamini adjustments for multiple testing (FDR 0.2 and 0.05). The groups did not differ in any of the other physical measures taken (Body condition score; Foot lesions; Comb lesions; Comb elevation, colour and size).

#### 3.3.3 Blood and faecal measures (SNP and TNP hens)

The mean values for each group’s blood physiology measures are summarised in Table 3. There were no significant differences between SNP and TNP birds. There were also no significant differences between SNP and TNP birds in faecal bulk water content.

#### 3.3.4 Behavioural observations (SNP and TNP hens)

**Fig. 3:**
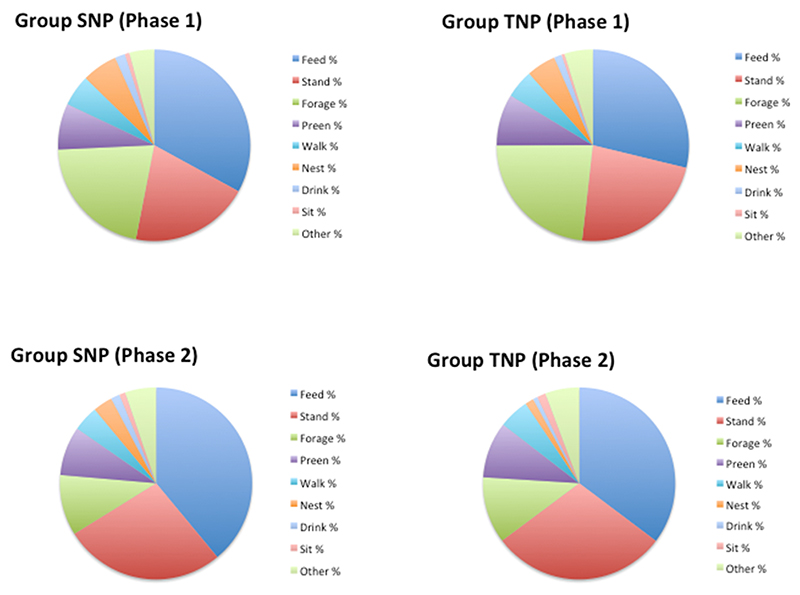
Pie charts illustrating the behavioural time budgets observed in SNP and TNP birds, during scan sample observations in Phases 1 and 2.

Fig. 3 shows the behavioural time-budgets of SNP and TNP between Phases 1 and 2, obtained from scan observations. It can be seen from these pie charts that the shift between Phases 1 and 2 (Intermediate to Non-Preferred living conditions) was accompanied by birds moving away from Foraging behaviours (Ground Peck and Ground Scratch) in both SNP and TNP hens. This is because the Non-Preferred living condition had wire flooring which has low compatibility with these natural behaviours. Analyses revealed no significant differences between the groups in the mean frequencies of scan-observed behaviours in the Phase 2 data collection period.

Analyses of less frequent behaviours observed during focal behavioural observations (i.e. behaviours occurring, on average, on <1 % of scans), revealed a number of significant differences between SNP and TNP birds. With respect to social behaviour, TNP birds, but not SNP birds, showed increases in Mild feather pecking and Aggressive attacking of pen-mates between Phases 1 and 2 (by Phase 2, 63 % of TNP and 43% of SNP birds showed Mild feather pecking of pen mates during focal observations: β = 1.384 (SE 0.642), p = 0.031; 40 % of TNP and 23 % of SNP birds were observed Aggressively attacking pen mates: β = 1.363 (SE 0.695), p = 0.050). The percentage of birds seen performing Head-shaking behaviour was also higher in TNP birds in Phase 2 (83 % of TNP birds and 53 % of SNP birds were observed Head-shaking: β = 1.443 (SE 0.616), p = 0.019). However, none of these differences persisted following Benjamini adjustments for multiple testing (FDR 0.2 and 0.05).

#### 3.3.5 Physical challenge tests measures (SNP and TNP hens)

Group SNP and TNP birds did not differ in their responses to the High Perch Test or the Water Box Test at Phase 2.

## 4 Discussion

The present study was designed to investigate whether birds experiencing improving (i.e. increasingly preferred, “positive trajectory”) living conditions show signs of more positive affect and better welfare than those experiencing stable, preferred conditions, and whether those experiencing worsening (i.e., increasingly less-preferred, “negative trajectory”) living conditions show signs of more negative affect and poorer welfare than those experiencing stable, non-preferred ones (Fig. 1).

We found that hens in Group TP (“Trajectory to Preferred”) did show some small differences from those in Group SP (“Stable Preferred”). TP birds showed more Foraging behaviour (Ground pecking and Ground scratching; remaining significant after Benjamini adjustments with FDRs of 0.2 and 0.05) and less Standing Alert (significant following Benjamini adjustments with an FDR of 0.2), both of which may be indicative of more positive affect and better welfare. Foraging on the ground is a natural behaviour that hens will perform for long periods even in the presence of ad libitum food supplied in a hopper; substrates including solid floors and wood shavings enable this behaviour, and it is seen more in preferred environments (Nicol et al., 2011; Paul et al., 2022). Standing Alert, on the other hand, is thought to be a component of vigilance behaviour, and can be seen at greater frequencies in less-preferred, more threatening environments (Nicol et al., 2009; Nicol et al., 2011); although (Paul et al., 2022) did not find differences in Standing Alert between hens living in generally preferred and non-preferred conditions). In sum, our hypothesis that an upward-trajectory of improving living conditions would result in evidence for more positive affect and better welfare (when compared with stable, preferred conditions) was not strongly supported, although we did find tentative evidence of some positive responsiveness to upward-change.

Group TNP (Trajectory to Non-Preferred) hens also showed some differences from Group SNP (Stable Non-Preferred) hens. They were more frequently observed doing Mild feather pecking and Aggressive attacking of pen mates, and also Head-shaking (Hughes, 1983); these may be indicative of somewhat poorer welfare due to greater social conflict resulting from reduced space, or frustration resulting from declining access to resources, in the downward-trajectory group (e.g., (Duncan and Wood-Gush, 1971) re: frustration). However, these differences were not substantial and did not remain significant following Benjamini adjustments for multiple testing (FDR 0.2 and 0.05). SNP hens had significantly longer claws than TNP ones (a potential welfare concern), but this difference can be simply explained by the longer time spent by SNP hens on wire pen flooring. Our hypothesis that a downward-trajectory of worsening living conditions would result in more negative affect and poorer welfare (when compared with stable, non-preferred conditions), therefore, was not convincingly supported.

Judgement bias tests revealed no significant differences between upward trajectory TP hens and stable SP hens, nor between downward trajectory TNP and stable SNP hens, in the extent to which they made “optimistic-like” or “pessimistic-like” decisions in response to ambiguous probe cues. The judgement bias task is generally considered to be both a valid and sensitive measure of affective state and thus welfare (Paul and Neville, in press). But, while judgement biases have frequently been found to be associated with affect manipulations, results have also been highly heterogeneous, with some null and contrary findings (Lagisz et al., 2020; Neville et al., 2020). Within studies of chicken welfare in particular, judgement bias tests have not always been effective at detecting group differences (e.g., Hernandez et al., 2015, Ross et al., 2019, Wichman et al., 2012). The chicken judgement bias task used in the present research, however, has a track record of good face validity and some responsiveness to affective manipulation (Paul et al., 2022; Deakin et al., 2016). It is possible, therefore, that the test simply lacked the sensitivity to differences between the experimental groups in this study. This may have been compounded by relatively low statistical power: in the present experiment it was only possible to judgement bias train and test 40 % of the whole study population (12 hens per experimental group). For example, it is possible that only a proportion of birds in each group experienced affective consequences from the trajectory manipulations; e.g., those higher in the social hierarchy in the TP group, and those lower in the social hierarchy in the TNP group, may have been particularly strongly affected. If this was the case, greater number of birds may have been needed to detect statistically significant differences between ‘trajectory’ and ‘stable’ groups.

Interpretations of small or near-null findings in research are inherently problematic, as one is unable to conclude that the hypothesised effects would not occur in any circumstances. For example, in the present case, it is possible that with a larger sample size, longer/shorter durations of stay in trajectory/non-trajectory living conditions,greater differences between the preferred and non-preferred conditions, and/or smoother/more abrupt transitions within trajectory conditions, responsiveness to trajectories of change in living conditions could have been more convincingly detected. It is also possible that alternative or additional measures of affective state or welfare, such as preference testing, may have been able to detect the hens’ responses that the current methods were unable to do. In our sister study (Paul et al., 2022), hens showed significant preferences for the SP condition after 24 weeks (labelled GP in (Paul et al., 2022). They also showed strong individual differences in living condition preference, which was significantly associated with judgement bias. Unfortunately, for experimental and logistical reasons, it was not possible to test the birds’ preferences for their living conditions at the end the 6-week period considered here (i.e., during Phase 2 data collection), so we cannot say whether preferences might have been stronger or weaker among the trajectory condition hens.

Another possibility is that the use of Intermediate living conditions during Phase 1, followed by drops down, or steps up, to the Preferred and Non-Preferred conditions at the start of Phase 2, may have dampened any subsequent effects of the trajectory shifts. This experimental design enabled all birds in the study to experience the same environments during habituation, training and initial (Phase 1) testing for the study. But it is possible that future research may offer more clear-cut results if, for example, downward shifting vs static Non-Preferred environments were compared in birds that had previously only experienced Preferred living conditions, including throughout the rearing period.

Of course, another possible explanation for our findings in this study is that hens’ affective states and welfare are not in fact influenced by changes in their living conditions. That is, although domestic chickens, like many other animals, can clearly form preferences and dispreferences about resources and environments (e.g. (Weeks and Nicol, 2006), show evidence of better or poorer welfare in differing living conditions (e.g., Nicol et al., 2009; Ross et al., 2019), and gradually learn about the changing values of reward and punishment that occur in their environment (Davies et al., 2015; Rescorla and Wagner, 1972; Schultz et al., 1997), they may not be *affectively sensitive* to (or *affectively responsive* to) such changes. In the introduction we cited a number of fields of evidence indicating that humans are affectively responsive to changes and trajectories of rewarding and punishing experiences. Unexpected events, whether disappointing or positive can have considerably greater impacts on people’s self-reported emotions than expected ones, and shifts in life circumstances can have greater affective impacts in the short-to medium term than can long-term exposure to stable conditions, whether good or bad (e.g., Diener et al., 2006; Easterlin, 2015; Frederick and Loewenstein, 1999; Luhmann et al., 2012; Rutledge et al., 2014; Rutledge et al., 2015; Shepperd and Mcnulty, 2002). We also suggested that there may be adaptive reasons for both humans and other animals to be sensitive to changes and trajectories of reward and punishment in the environment (e.g., increasing/decreasing threats as predator numbers boom and bust; increasing/decreasing food availability as seasons change). Specifically, if an individual is affectively sensitive to such changes, they may be better able to guide appropriate decision-making in situations of novelty or ambiguity (Mendl and Paul, 2020; McNamara et al., 2013).

Currently, there is experimental evidence to show that a number of non-human species are affectively responsive to changes in levels of reward and punishment, at least within short-term timescales. Most notably, successive negative contrast tasks (Crespi, 1942; Flaherty, 1996; Papini and Dudley, 1997) have demonstrated that a number of mammals show disappointment-like responses to sudden drops in the value of expected rewards (e.g., (Bentosela et al., 2009) - dogs; (Mustaca et al., 2000) - mice; (Papini et al., 1988) – opossums). In a typical paradigm, animals (e.g., rats) that are trained to run along a runway for a food reward, run more slowly when the reward diminishes suddenly than do other animals that have been rewarded with this smaller amount of food all along (e.g., (Burman et al., 2008b). This slowed running is interpreted as a relative lowering of reward expectation – i.e., a reduced willingness to work (run) for the poorer reward. However, in a number of non-mammalian species, including chickens, these sorts of contrast effects have not been found (Couvillon and Bitterman, 1985) – goldfish; (Davies et al., 2015) – chickens; (Papini and Ishida, 1994) - turtles; (Pelegrini et al., 2008) – pigeons; (Petherick et al., 1990) - chickens; (Schmajuk et al., 1981) – toads; (Tan et al., 2020) - zebrafish). It is possible that methodological problems may have been involved in these null findings (e.g., see Davies et al., 2015; Riemer et al., 2016). But it is also possible that they indicate a broad lack of affective responsiveness to change and contrast in reward value, akin to disappointment or frustration, in chickens and perhaps other non-mammalian species (although see Freidin et al., 2009) and (Schnell et al., 2021) for evidence of affective responses to contrast in some other species of birds). If this is the case, then it is also possible that changes in living conditions, such as those considered in the present study, may not have any direct affective impact on these species.

## Conclusion

We conclude that the hens in the present study showed some evidence of responsiveness to ‘affective trajectories’ in their living conditions, but no definitive effects on their affective states and welfare. Nevertheless, we suggest that trajectory (and other contrast) manipulations remain worthy of future investigation, both for the purposes of a developing understanding of the processes involved in animal affect, and for offering potentially novel and effective solutions for improving the welfare of domestic and captive animals. Future studies of affective trajectories such as those hypothesised here may be more successful at discovering affective and welfare differences in response to trajectories of preferred and non-preferred environments if they make use of more refined methodologies than those used here. For example, using larger samples in which data on dominance and subordinance can be incorporated into the analyses may prove invaluable. And using control groups who experience changes in their living conditions (e.g., zig-zagging back and forth between Preferred and Non-Preferred environments) would enable researchers to disentangle the respective effects of *change* and *trajectory of change*.

Finally, focusing on mammalian species, in whom short-term affective sensitivity to changing reward has been observed (e.g., see (Papini and Dudley, 1997) for review), may also increase the chances of detecting sensitivity to affective trajectories in living conditions. Species that have previously shown affective responsiveness to changes in environmental enrichment may also be good candidates for such research (e.g., rats – (Barker et al., 2017); (Brydges et al., 2011).

## Supplementary Material

Supplementary material

Appendix A

## Figures and Tables

**Table 1:** Housing conditions and pen furniture for each of the living conditions, I (Intermediate), NP (Non-preferred) and P (Preferred), and for each step between NP and P. Group TP experienced NP, NP1, NP2, P2, P1, P successively in Phase 2, while Group TNP experienced P, P1, P2, NP2, NP1, NP successively in Phase 2. Positive events: *Sweetcorn, spaghetti, cheese, heated pad*. Negative events: *Water spray, inflatable toy, alarm call played on loud-speaker, dog bark played on loud-speaker*. Floor space per bird in living condition P was 0.75 m^2^ per bird, and in living condition NP it was 0.37 m^2^ per bird. Perches in living condition P were double-tiered and provided birds with 40 cm of space each. Perches in living condition NP were single tiered and provided birds with only 8 cm of space each (i.e., competition was inevitable). Within the laboratory, two P pens, or four NP pens, were each experimental room.

			Living condition							
	Resource		I(All hens in Phase 1)	NP (Group SNP hens in Phase 2)	NP1	NP2	P2	P1	P (Group SP hens in Phase 2)	
	**Pen Size (m)**		1.22 × 2.25	1.22 × 1.52	1.22 × 1.52	1.22 × 2.25	1.22 × 2.25	1.22 × 3.06	1.22 × 3.06	
	**Pen Floor**		Shavings (4 cm)	Wire mesh	Wire mesh	Shavings (4 cm)	Shavings (4 cm)	Shavings (10 cm)	Shavings (10 cm)	
	**Perches**		L: 60 cm H: 15 cm	L: 40 cm H: 5 cm	L: 40 cm H: 15 cm	L: 60 cm H: 15 cm	L: 60 cm H: 15 cm	L: 100 cm H: 30 cm	L: 100 cm H: 30 cm L: 100 cm H: 60 cm	
	**Nest Box (300 mm wide, 300mmm** ** deep, 830 mm tall)**		Frame Roof Base Tier Sides Back	Frame Roof	Frame Roof Base Tier	Frame Roof Base Tier Sides Back	Frame Roof Base Tier Sides Back Perch	Frame Roof Base Tier Sides Back Perch Mat	Frame Roof Base Tier Sides Back Perch Mat Astroturf	
	**Dust Bath**		Yes Shavings (4 cm)	No	No	Yes Shavings (4 cm)	Yes Shavings (10 cm)	Yes Shavings (10 cm)	Yes Sand and compost (10 cm)	
	**Shade (black fabric cover above** ** perch to restrict light) Ambient noise**		None Background (50 dB)	None White noise (80 dB: 4 h/day)	None White noise (75 dB: 4 h/day)	None White noise (70 dB: 4 h/day)	Half perch Background (50 dB)	Whole perch Background (50 dB)	Whole perch Background (50 dB)	
	**Positive Events**		None	None	None	None	1 per day	2 per day	4 per day	
	**Negative** ** Events**		None	4 per day	2 per day	1 per day	None	None	None	

**Table 2:** Mean body mass and mean blood concentrations of corticosterone, glucose, heterophil/lymphocyte ratios and triglycerides for SP and TP hens (*standard deviations in itallics*).

Measure	Phase 1			Phase 2		
	SP	TP		SP	TP	
Body Mass (g)	1740.47	1690.43		1815.53	1724.69	
* s.d.*	*135.02*	*142.16*	* *	*145.16*	*124.44*	* *
Corticosterone (ng/ml)	2.46	2.71		2.29	3.09	
* s.d.*	*1.22*	*1.51*	* *	*1.40*	*1.83*	* *
Glucose (mmol/l)	13.99	13.70		13.10	13.45	
* s.d.*	*0.82*	*0.96*	* *	*0.61*	*1.15*	* *
H/L ratio	1.27	1.72		1.82	1.96	
* s.d.*	*0.51*	*1.59*	* *	*1.36*	*1.79*	* *
Triglycerides (mmol/l)	14.71	12.33		14.54	12.88	
* s.d.*	*6.12*	*4.67*	* *	*8.03*	*5.69*	* *

**Table 3:** Mean body mass and mean blood concentrations of corticosterone, glucose, heterophil/lymphocyte ratios and triglycerides for SNP and TNP hens (*standard deviations in itallics*).

Measure	Phase 1			Phase 2		
	SNP	TNP		SNP	TNP	
Body Mass (g)	1761.80	1685.47		1794.37	1741.67	
* s.d.*	*136.17*	*173.98*	* *	*143.49*	*176.56*	* *
Corticosterone (ng/ml)	2.90	2.63		2.32	2.03	
* s.d.*	*1.66*	*1.84*	* *	*1.19*	*1.07*	* *
Glucose (mmol/l)	13.67	13.89		13.16	13.08	
* s.d.*	*1.31*	*1.21*	* *	*0.82*	*0.69*	* *
H/L ratio	1.23	1.61		1.43	1.85	
* s.d.*	*0.60*	*1.15*	* *	*0.98*	*1.41*	* *
Triglycerides (mmol/l)	13.32	12.03		17.32	15.10	
* s.d.*	*4.09*	*3.66*	* *	*9.16*	*6.00*	* *
