# Supplementary material for: Affective trajectories: Are hens influenced by positive and negative changes in their living conditions?
Source: Appl Anim Behav Sci. Author manuscript; Available in PMC 2024 Jul 31. (PMC7616324; doi:10.1016/j.applanim.2023.105883)
Supplement: Appendix A [file EMS197739-supplement-Appendix_A.pdf]

Appendix A. Supporting information

Supplementary data associated with this article can be found in the online version at doi:10.1016/j.applanim.2023.105883.
